# Aquaporin-mediated increase in root hydraulic conductance is involved in silicon-induced improved root water uptake under osmotic stress in *Sorghum bicolor* L.

**DOI:** 10.1093/jxb/eru220

**Published:** 2014-05-30

**Authors:** Peng Liu, Lina Yin, Xiping Deng, Shiwen Wang, Kiyoshi Tanaka, Suiqi Zhang

**Affiliations:** ^1^State Key Laboratory of Soil Erosion and Dryland Farming on the Loess Plateau, Institute of Soil and Water Conservation, Chinese Academy of Sciences and Ministry of Water Resources, Yangling, Shaanxi, 712100, China; ^2^Institute of Soil and Water Conservation, Northwest A&F University, Yangling, Shaanxi, 712100, China; ^3^Faculty of Agriculture, Tottori University, Koyama Minami 4–101, Tottori 680-8533, Japan

**Keywords:** Aquaporin, osmotic stress, root hydraulic conductance, silicon, transpiration rate, whole-plant hydraulic conductance.

## Abstract

The fact that silicon application alleviates water deficit stress has been widely reported, but the underlying mechanism remains unclear. Here the effects of silicon on water uptake and transport of sorghum seedlings (*Sorghum bicolor* L.) growing under polyethylene glycol-simulated osmotic stress in hydroponic culture and water deficit stress in sand culture were investigated. Osmotic stress dramatically decreased dry weight, photosynthetic rate, transpiration rate, stomatal conductance, and leaf water content, but silicon application reduced these stress-induced decreases. Although silicon application had no effect on stem water transport capacity, whole-plant hydraulic conductance (K_plant_) and root hydraulic conductance (Lp) were higher in silicon-treated seedlings than in those without silicon treatment under osmotic stress. Furthermore, the extent of changes in transpiration rate was similar to the changes in K_plant_ and Lp. The contribution of aquaporin to Lp was characterized using the aquaporin inhibitor mercury. Under osmotic stress, the exogenous application of HgCl_2_ decreased the transpiration rates of seedlings with and without silicon to the same level; after recovery induced by dithiothreitol (DTT), however, the transpiration rate was higher in silicon-treated seedlings than in untreated seedlings. In addition, transcription levels of several root aquaporin genes were increased by silicon application under osmotic stress. These results indicate that the silicon-induced up-regulation of aquaporin, which was thought to increase Lp, was involved in improving root water uptake under osmotic stress. This study also suggests that silicon plays a modulating role in improving plant resistance to osmotic stress in addition to its role as a mere physical barrier.

## Summary

This study demonstrated that silicon-enhanced root hydraulic conductance through up-regualtion of aquaporin gene expression resulted in improved root water uptake under osmotic stress in sorghum.

## Introduction

Silicon is the second most abundant element in the soil after oxygen, and comprises 31% of the earth’s crust (Epstein, 1999). Although silicon is not currently considered an essential element for higher plants, silicon uptake has frequently been found to be beneficial in increasing plant resistance to multiple stresses (Ma, 2004; Guntzer, 2011), including pests and pathogens (Garbuzov *et al.*, 2011; Dallagnol *et al.*, 2012), metal toxicity (Rizwan *et al.*, 2012), and salt and water stress (Hattori *et al.*, 2005; Gong *et al.*, 2006). Although the effects of silicon on plant resistance of such stresses have been well identified, the mechanism underlying silicon’s capacity to increase stress resistance is still poorly understood.

Numerous studies have shown that silicon is effective in improving plant drought resistance in wheat, sorghum, maize, soybean, and rice (Gong *et al.*, 2005; Hattori *et al.*, 2005; Gao *et al.*, 2006; Shen *et al.*, 2010; Nolla *et al.*, 2012). The mechanisms involved include decreasing the transpiration rate and maintaining water content through silica deposits in the leaf cuticle, as seen in rice (Matoh *et al.*, 1991); adjusting the osmotic potential through changes in the accumulation of proline, inorganic ions, and other osmotic solutes, as seen in sorghum and rice (Sonobe *et al*., 2010; Ming *et al.*, 2012); minimizing drought-induced oxidative damage through regulating enzymatic and non-enzymatic antioxidant capacities (Shen *et al.*, 2010); and enhancing the activities of photosynthetic enzymes, such as ribulose bisphophate carboxylase and NADP^+^-dependent glyceraldehyde-3-phosphate dehydrogenase (Gong *et al.*, 2005). Most previous studies have concluded that silicon improves drought resistance through decreasing water loss or oxidative damage.

Sorghum is one of the world’s most important crops, and silicon application could improve its drought resistance (Hattori *et al.*, 2005; Sonobe *et al*., 2010). Previous research has indicated that silicon enhances drought resistance through decreasing the plant transpiration rate, which leads to a reduction in water loss in rice and maize (Matoh *et al.*, 1991; Gao *et al.*, 2006). In sorghum, a similar gramineous plant, however, the transpiration rate is enhanced by silicon, and leaf water content under drought stress is higher in the presence than in the absence of applied silicon (Hattori *et al.*, 2005). This implies that silicon’s improvement of sorghum drought resistance is probably related to increases in water uptake or transport, but not to a reduction in water loss as in other gramineous species. However, the mechanism by which silicon regulates water uptake and transport under drought stress has been largely ignored.

In plants, the overall water transport is represented by whole-plant hydraulic conductance (K_plant_), which consists of leaf, stem, and root hydraulic conductance (Martre *et al.*, 2002). In leaves, hydraulic conductance (K_leaf_) is coordinated with leaf water potential (Brodribb *et al*., 2003). In stems, hydraulic conductance, usually represented as leaf-specific conductivity (L_sc_), is mainly controlled by vessel characteristics and embolism (Lovisolo *et al.*, 1998). In roots, hydraulic conductance represents water uptake capacity, and mainly depends on the driving force, root surface, root anatomy, and root water permeability (Steudle, 2000b; Vandeleur *et al.*, 2009; Sutka *et al.*, 2011). The dominating driving force for water uptake is hydrostatic forces (i.e. pressures or tensions). In addition, the osmotic gradient may also be beneficial in water uptake (Javot and Maurel, 2002). Root surface and anatomy play an important role in regulating the apoplastic pathway of water uptake. Root water permeability could be regulated by aquaporin, which is thought to regulate root water uptake especially under drought stress (Steudle, 2000b; Maurel *et al.*, 2008).

Upon long-term (>3 d) exposure to drought stress, roots can respond with marked surface and anatomical alterations, which in turn cause profound changes in their water transport capacity; however, before any changes in root surface and anatomy can be observed, the water permeability is already changed to regulate water uptake capacity in the root (Javot and Maurel, 2002). In sorghum subjected to 3 weeks of drought stress, silicon does not affect the root surface area or anatomical characteristics, but it does increase the transpiration rate (Sonobe *et al*., 2010). Furthermore, in a preliminary experiment, in which sorghum was subjected to short-term (<3 d) osmotic stress, the transpiration rate was also higher in silicon-treated seedlings than in untreated seedlings. Therefore, it was speculated that silicon reduces the decrease in sorghum transpiration rate and that the relative water content (RWC) is involved in the observed changes in K_plant_, especially the root hydraulic conductance (Lp), under short-term osmotic stress.

The purpose of this study was to test the hypothesis that silicon improves water uptake by reducing the decrease in plant hydraulic conductance, and thereby enhances sorghum resistance to osmotic stress. To accomplish this objective, K_plant_, L_sc_, Lp, and the function of aquaporin were measured in sorghum seedlings grown in both hydroponic and sand cultures. In hydroponic culture, it is easy to obtain intact roots to measure Lp and aquaporin function, whereas sand culture promotes stem growth and facilitates the measurement of L_sc_.

## Materials and methods

### Plant material and growth conditions

Seedlings of sorghum [*Sorghum bicolor* (L.) Moench. cv. Gadambalia] were cultivated in a growth chamber under cycles consisting of 14h of light (450 μmol m^−2^ s^−1^) at 28 °C and 10h of darkness at 23 °C. The relative humidity was 40–50%.

### Experiment 1: the effects of silicon on sorghum resistance to osmotic stress, whole-plant hydraulic conductance, root hydraulic conductance, and aquaporin in hydroponic culture

#### Seedling cultivation, silicon, and polyethylene glycol (PEG) treatment 

Sterilized seeds were germinated for 4 d in an incubator at 25 °C. After germination, healthy seedlings were transplanted into a plastic container with 8 litres of one-quarter strength Hoagland culture solution. After 6 d, the culture solution was changed to half strength. After a total of 9 d after transplanting, the culture solution was changed to full strength. For silicon treatment, 0mM or 1.67mM Na_2_SiO_3_ was added to the culture solution starting the third day after transplanting. The culture solution was continuously aerated, and the pH was adjusted to 6.0 with 0.1M HCl or 1M KOH every day. Twelve days after transplanting, 10% PEG-6000 (–0.2MPa) was added at 08:00h to induce osmotic stress, and, unless stated otherwise, samples were collected and measurements were made from 10:00h to 13:00h on this day.

#### Biomass and silicon concentration determination 

The dry weight of sorghum seedlings was measured after 3 d and 7 d of PEG treatment. The silicon concentration in plant materials was determined according to Van der Vorm (1987). Briefly, the shoot and root were sampled and dried at 75 °C for 72h. Dried powder of plant materials was ashed in porcelain crucibles at 550 °C for 3h, and then extracted by 0.08M H_2_SO_4_ and 40% HF. The silicon concentration was determined by the colorimetric molybdenum blue method at 811nm. The silicon concentration was expressed as μmol g^–1^ dry weight.

#### Photosynthetic rate, stomatal conductance, and transpiration rate 

The photosynthetic rate, stomatal conductance, and transpiration rate were measured with a portable photosynthesis system (Li-6400; LI-COR Inc., Lincoln, NE, USA). The new fully expanded leaf was placed in a chamber at a photon flux density of 500 μmol m^–2^ s^–1^; the flow rate through the chamber was 500 μmol s^–1^ and leaf temperature was 28 °C. The leaves were typically 1.7cm wide and the area in the chamber was determined for each leaf. Transpiration rates used to calculate K_plant_ were also determined gravimetrically in the first 2h from the onset of PEG treatment. Each treatment includes five replications.

#### Leaf relative water content and water potential 

The leaf RWC was measured according to Machado *et al.* (2001). Ten leaf discs (9mm in diameter) from fully expanded leaves were weighed immediately for measurement of fresh weight (FW). The discs were floated in distilled water for 6h, then dried with filter paper and weighed for measurement of total weight (TW). Dry weight (DW) was measured after drying the discs at 70 °C in a forced-air oven for 24h. The relative water content was calculated as:

RWC=[(FW–DW)/(TW–DW)]×100

New fully expanded leaves were covered with aluminium foil prior to excision from the plant. The water potential was measured by using a pressure chamber (Model 3500, Soilmoisture Corp., Santa Barbara, CA, USA). Each treatment includes five replications.

#### Osmotic potential of root xylem sap 

The osmotic potential of the root xylem sap was measured according to the method of Kaufmann and Eckard (1971). Each shoot was cut off at the base of the root system leaving 4cm of mesocotyl. The mesocotyl was sealed with silicon seals which had a hole adjusted to the diameter of the mesocotyl. The xylem sap was force exuded by N_2_ pressurized to 2 bar. About 15 μl of root xylem sap was collected and sealed in a microtube. The osmotic potential of the collected sap was determined using a vapour pressure osmometer (Model 5520, Wescor, Logan, UT, USA). Each treatment includes five replications.

#### Whole-plant hydraulic conductance (K_plant_) 

The K_plant_ was calculated according to the following equation (Martre *et al.*, 2002):

$${\text{K}}_{\text{plant}}=\text{Transpiration rate}/\left(\text{Soil water potential}-\text{Leaf water potential}\right)$$

The transpiration rate was determined gravimetrically. The leaf water potential was measured as mentioned above. In hydroponic culture, the soil water potential (i.e. culture potential) was –0.07MPa or –0.09MPa under control conditions or with silicon application, respectively. Under PEG treatment, the soil water potential (i.e. culture potential) was –0.2MPa. This experiment includes five replications in each treatment.

#### Root hydraulic conductance (Lp) and root surface area 

The Lp based on the root surface area was measured with a pressure chamber according to the method of Miyamoto *et al.* (2001). Each shoot was cut off at the base of the root system leaving 4cm of mesocotyl. The mesocotyl was sealed with silicon seals which had a hole adjusted to the diameter of the mesocotyl. The pressure in the chamber was raised in steps of 0.1MPa up to 0.5MPa. Exuded sap was collected with absorbent cotton and weighed. For a given gas pressure, the volume exuded from the root system was plotted against time. The slopes of these relationships referred to the unit root surface area. This yielded the volume flow, Jvr in m^3^ m^–2^ s^–1^. Root Lp is calculated from the slopes of Jvr against driving force, and the driving force consisted of Pgas and osmotic gradient. In this study, however, the osmotic driving force was too small to be considered; as a result, Lp was determined from the slopes of Jvr against Pgas only, according to the following equation:

Jvr=Lp×Pgas

After the exuded sap was measured, the root was sampled to determine root surface area using a scanner and analysed by WinRHIZO PRO 2009 software (Regent Inc., Canada). Root surface area was calculated from projected areas of root that were assumed to be cylindrical in shape. Each treatment includes five replications.

#### Transpiration rate responds to aquaporin inhibitor (HgCl_2_) and dithiothreitol (DTT) 

According to the method of Knipfer *et al.* (2011), aquaporin-mediated water transport was investigated by measuring changes in the transpiration rate in response to the application of the aquaporin inhibitor HgCl_2_. One group of seedlings was used for measuring the transpiration rate. The other group was treated with 50 μM HgCl_2_ for 5min and subsequently rinsed with distilled water before being returned to the culture solution (devoid of HgCl_2_) where the transpiration rate was measured again. The reversibility of the effect of HgCl_2_ on aquaporin activity was tested by treating roots first with 50 μM HgCl_2_ and then placing them for 15min in 5mM DTT before measuring the transpiration rate. In addition, to confirm further the participation of aquaporin in silicon-induced water transport, NaN_3_, another widely used aquaporin inhibitor, was also applied in this study in the same manner as HgCl_2_.

#### Expression analysis of sorghum aquaporin genes 

Root tips 3cm in length were collected after 4h and 24h PEG treatment and frozen in liquid nitrogen to measure the expression of aquaporin genes. Eight sorghum plasma membrane intrinsic protein (*SbPIP*) aquaporin genes were identified based on data from the NCBI. The genes, as well as the sequences of their specific primers, are presented in Table 1. DNA sequence comparisons were made to ensure that each pair of primers was specific to the corresponding *SbPIP* gene.

**Table 1.** The primers of the *SbPIP* aquaporin genes and reference gene

Gene ID	Gene	Primer	Product size (bp)
Sb01g010030	*Actin1*	F 5′-TGTTCCCTGGGATTGCTG-3′ R 5′-GCCGGACTCATCGTACTCA-3′	185
Sb06g025150	*PIP1;3/1;4*	F 5′-AATCGGGTTCGCGGTGTT-3′ R 5′-CCAGGCATGGTTCTGGTTGTA-3′	115
Sb04g032430	*PIP1;3/1;4 (2)*	F 5′-GTGGAGCTGGAGTGGTGAA-3′ R 5′-GCAAGGATAGGAACATGGGAGT-3′	199
Sb04g037800	*PIP1;5*	F 5′-TTTCGCCGTCTTCCTCGTC-3′ R 5′-GGTCGTTCCATGCGTTGG-3′	116
Sb10g007610	*PIP1;6*	F 5′-TGACGGTGCTGACGGTGAT-3′ R 5′-GGAGGAGCCCGAAGGTGAC-3′	168
Sb02g010760	*PIP2;2*	F 5′-GACTCCCACGTCCCGGTTCT-3′ R 5′-CCCAGGGCTTGTCCTTGTTGT-3′	148
Sb04g026650	*PIP2;3*	F 5′-CCGTGACCTTCGGTTTGTTC-3′ R 5′-GCACGTAGTAGGCGCTCTGG-3′	132
Sb06g022840	*PIP2;5*	F 5′-TCGCGGTGTTCATGGTCC-3′ R 5′-TCCCAGGTCTTGTCGTTGTTGT-3′	109
Sb02g010800	*PIP2;6*	F 5′-CTTCCGATTGGATTCGCTGTG-3′ R 5′-CGGAGGACGATCTGGTGGTA-3′	197

Total RNA was extracted from 100mg of frozen root samples using an RNeasy^®^ Plant Mini Kit (Qiagen, Hilden, Germany) according to the manufacturer’s instructions, and treated with recombinant DNase I (RNase-free; Takara Bio, Shiga, Japan) to remove the remaining genomic DNA. Reverse transcription was performed using an iScript™ cDNA Synthesis Kit (Bio-Rad, Hercules, CA, USA) according to the manufacturer’s instructions. The cDNA was then diluted 50-fold in water, and 2 μl of cDNA was used to carry out the quantitative RT-PCR (qRT-PCR). The iQ™ SYBR^®^ Green Supermix (Bio-Rad) was used for the qRT-PCR on a MiniOpticon™ CFD-3120J1 instrument (Bio-Rad). A melting curve analysis was performed to confirm the absence of multiple products and primer dimers. To confirm further that the single peaks from the melting analysis corresponded to a unique amplification product of the correct size, the PCRs were run on a 1% agarose gel (data not shown). Data acquisition and analysis of qRT-PCR was done using Bio-Rad CFX manager software (version 2.0), and the expression levels of target genes were normalized to that of the internal control gene *Actin1* using the 2^ΔCt^ method. Each treatment includes three replications and each replication includes two technical replications.

### Experiment 2: the effects of silicon on leaf-specific conductivity of the stem in sand culture

#### Seedling cultivation, and silicon and water deficit treatment 

Seeds were sterilized with 1% sodium hypochlorite for 15min and then washed with distilled water four times. After sterilization, seeds were placed on damp filter paper in a Petri dish and germinated for 1 d in an incubator at 25 °C. After germination, five seeds were sown in a plastic pot which was filled with 8kg of sand that had been washed with distilled water three times to eliminate the effect of any soluble silicon in the sand. Twelve days after sowing, the seedlings were thinned to two seedlings per pot. During the growth period, the soil water content was controlled at 0.08g g^–1^ by watering with Hoagland solution. Starting after 30 d of growth, seedlings were watered with Hoagland solution containing 0mM or 1.67mM Na_2_SiO_3_. Starting after 38 d, half of the seedlings (including some silicon-treated and some untreated) were left unwatered until the soil water content had decreased to 0.03g g^–1^; the pot was weighed continuously, and the soil water content was calculated according to the weight (Hattori *et al.*, 2005). This water content was reached after 2 d and was maintained for another 1 d. At a soil water content of 0.03g g^–1^, the plants showed obvious water deficit stress: the leaves showed signs of dehydration and the photosynthetic rate decreased by 35–50%. In order to compare the results obtained with those from the hydroponic culture, the photosynthetic rate, stomatal conductance, and transpiration rate were also measured.

#### Leaf-specific conductivity of thestem 

The leaf-specific conductivity of the stem (L_sc_) was measured according to the method of Meinzer *et al.* (1992). All of the experiments were conducted between 10:00h and 13:00h. The stem was excised near the soil surface in the growth chamber and transported to the laboratory quickly (within 1min to minimize the effect of cavitation); there, the leaf was excised from the stem and saved for later measurement of the leaf area above the node. The stem was recut under water to keep the nodal segment 5cm long. At this growth stage of sorghum, the length of the node is ~1cm while that of the internode is ~4cm. Thus, the segment being measured consisted of 2cm of upper internode, 1cm of node, and 2cm of lower internode. The segment was inserted into the pressure chamber, which was sealed with silicon seals. Flow induced by a 0.025MPa pressure difference was measured over two successive 3min intervals. The L_sc_ of the stem was calculated as the flow rate divided by the pressure gradient along the segment and the leaf area above the node. Each treatment includes five replications and each replication includes two seedlings.

### Statistical analysis

Data were subjected to analysis of variance (ANOVA) using Statistical Analysis System (SAS version 8.0) software. Differences between the means were compared by means of the Tukey–Kramer test at *P*<0.05. All experiments were repeated at least twice.

## Results

### Biomass and silicon concentration

**Fig. 1.**
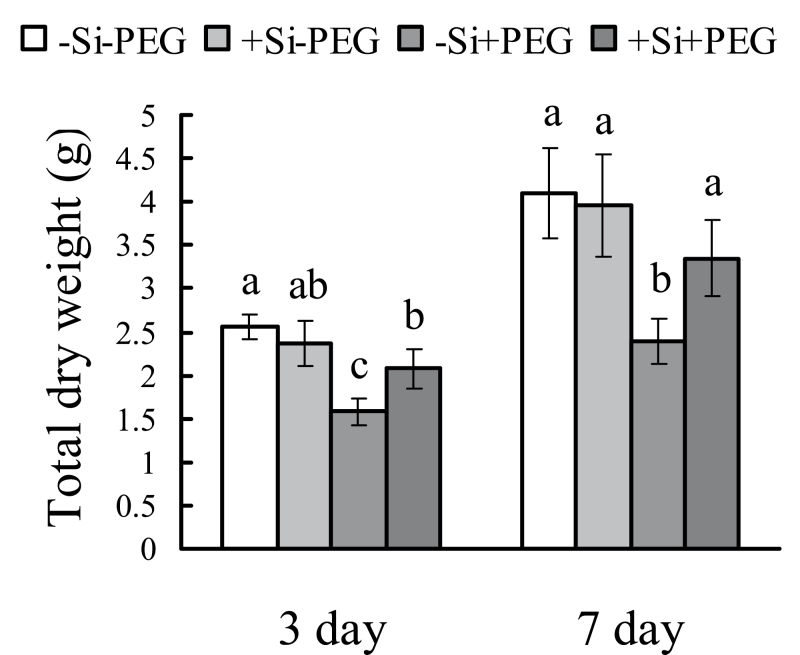
Effects of silicon application and osmotic stress on total dry weight of seedlings in hydroponic culture. The total dry weight of seedlings was investigated after 3 d and 7 d of exposure to osmotic stress. Values are means ±SD of five replicates. Different letters indicate a significant difference (*P*<0.05).

**Fig. 2.**
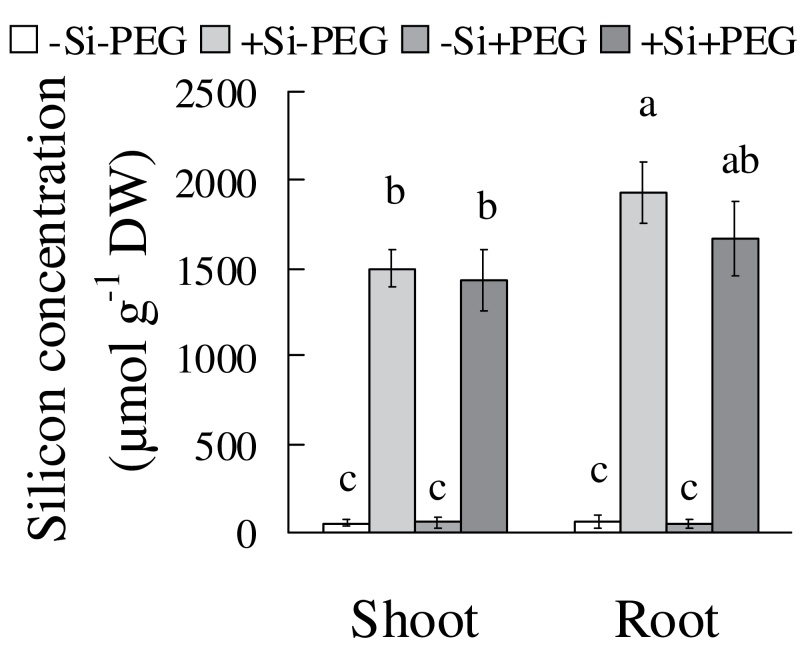
Effects of silicon application and osmotic stress on shoot and root silicon concentration. The dried powder of plant materials was ashed in porcelain crucibles at 550 °C for 3h, and then extracted with 0.08M H_2_SO_4_ and 40% HF. The silicon concentration was determined by the colorimetric molybdenum blue method at 811nm. Values are means ±SD of five replicates. Different letters indicate a significant difference (*P*<0.05).

Under control conditions, the total dry weights of silicon-treated and untreated seedlings were not different. Under osmotic stress, the total dry weights of untreated seedlings decreased by 38% and 42% after 3 d and 7 d of osmotic stress, while those of silicon-treated seedlings decreased by only 12% and 15%, respectively (Fig. 1). Silicon concentration was far higher in silicon-treated plants than in untreated plants in both shoot and root. PEG-6000 treatment did not affect silicon concentration in sorghum (Fig. 2).

### Photosynthetic rate, stomatal conductance, and transpiration rate

**Fig. 3.**
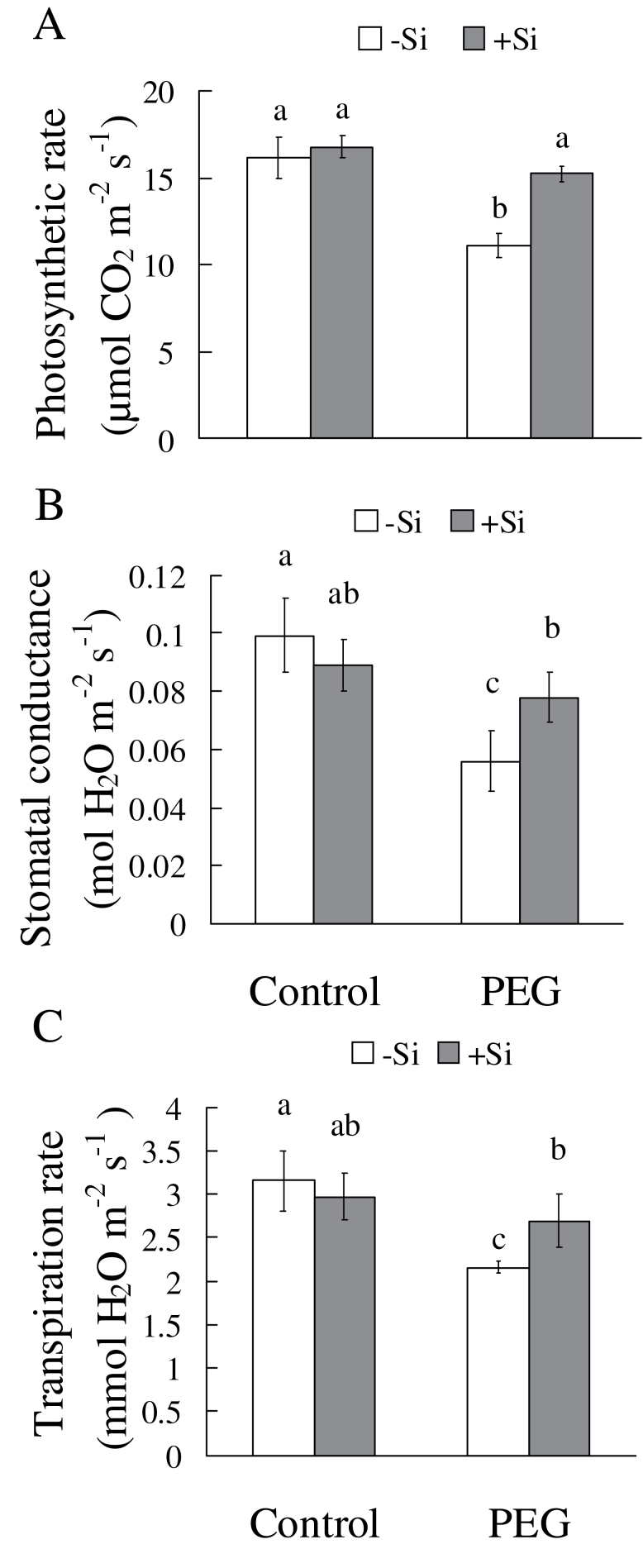
Effects of silicon application and osmotic stress on photosynthetic rate (A), stomatal conductance (B), and transpiration rate (C) in hydroponic culture. New fully expanded leaves were used for measurement in a portable photosynthesis system (Li-6400) after 2h osmotic treatment. Values are means ±SD of five replicates. Different letters indicate a significant difference (*P*<0.05).

Under control conditions, the leaf photosynthetic rate was not affected by silicon (Fig. 3A). When plants were exposed to osmotic stress, however, a significant decrease in photosynthetic rate was observed and silicon application reduced this decrease. Similarly, silicon had no effect on leaf stomatal conductance under control conditions, but, under osmotic stress, the stomatal conductance was 39% higher in silicon-treated seedlings than in untreated seedlings (Fig. 3B). Silicon treatment also caused no change in leaf transpiration rate under control conditions, whereas under osmotic stress the leaf transpiration rate was 25% higher in silicon-treated seedlings than in untreated plants (Fig. 3C). Whole-plant transpiration rate was also higher in silicon-treated seedlings than in untreated seedlings after as early as 20min of PEG treatment (Supplementary Fig. S1 available at *JXB* online). In addition, silicon showed a similar effect on changes in photosynthetic rate, stomatal conductance, and transpiration rate of sorghum seedlings grown under sand culture (Supplementary Fig. S2). These results show that silicon could reduce the decrease of photosynthetic rate, stomatal conductance, and transpiration rate under osmotic/water deficit stress in sorghum seedlings.

### Leaf relative water content, leaf water potential, and osmotic potential of root xylem sap

**Fig. 4.**
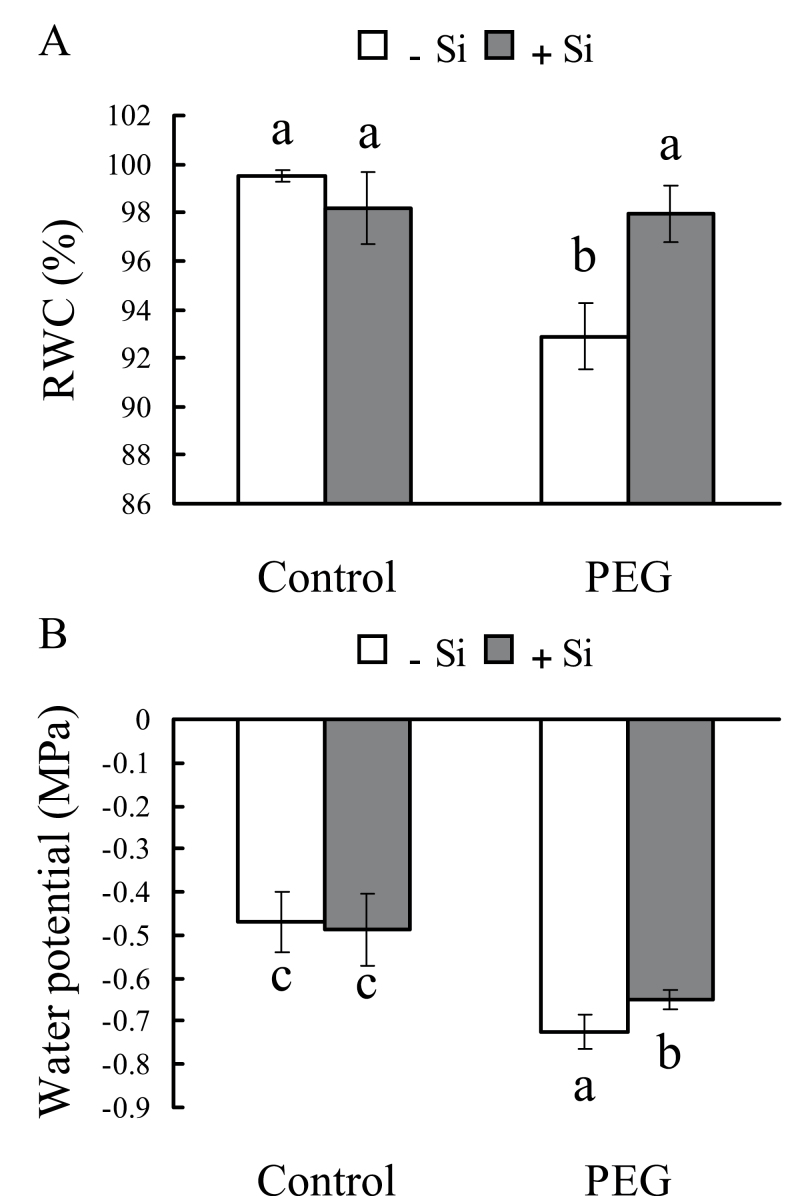
Effects of silicon application and osmotic stress on leaf relative water content (RWC) (A) and water potential (B) in hydroponic culture. After 2h osmotic treatment, 10 discs were cut from the leaves to investigate RWC, and leaf water potential was measured in a pressure chamber. Values are means ±SD of five replicates. Different letters indicate a significant difference (*P*<0.05).

**Fig. 5.**
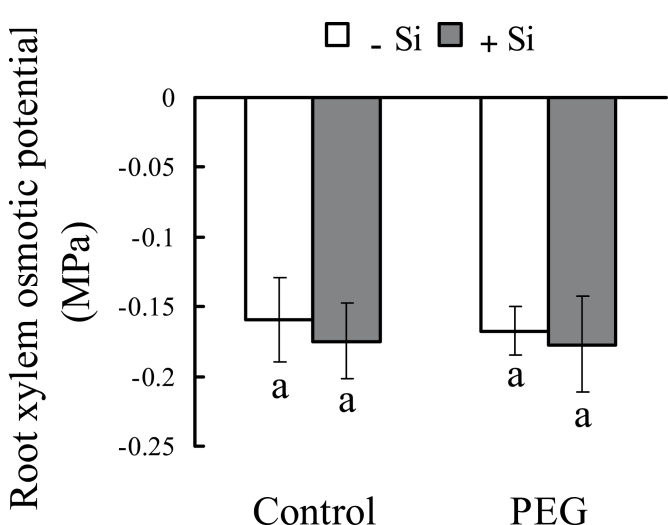
Effect of silicon application and osmotic stress on osmotic potential of root xylem sap in hydroponic culture. After 2h osmotic treatment, root xylem sap was collected and used for measurement of osmotic potential. Values are means ±SD of five replicates. Different letters indicate a significant difference (*P*<0.05).

Under control conditions, RWC was not affected by silicon in the present study (Fig. 4A). Under osmotic stress, although RWC was decreased, it was also higher in silicon-treated seedlings than in untreated seedlings. Under control conditions, the leaf water potential was about –0.5MPa both with and without silicon treatment (Fig. 4B). Under osmotic stress, it decreased to –0.72MPa without silicon treatment, but only to –0.65MPa with silicon treatment. These results suggest that silicon could reduce the decreases in RWC and leaf water potential that are caused by osmotic stress. In the present study, the osmotic potential of root xylem sap was not affected by short-term osmotic and/or silicon treatments (Fig. 5).

### Whole-plant hydraulic conductance

**Fig. 6.**
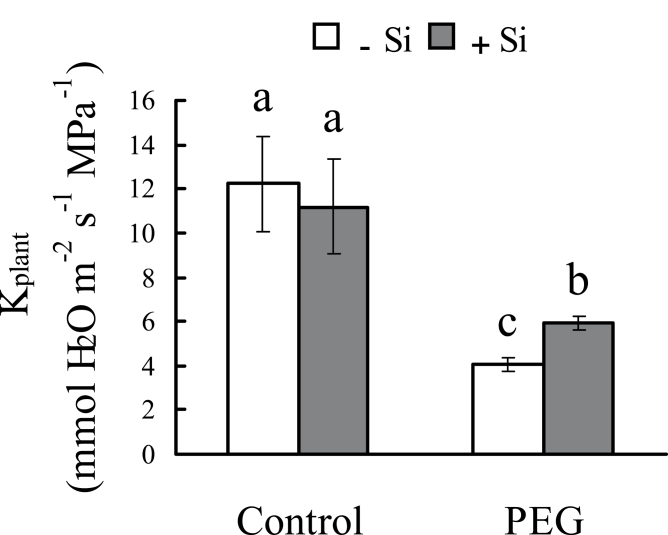
Effect of silicon application and osmotic stress on the whole-plant hydraulic conductance (K_plant_) in hydroponic culture. The K_plant_ was calculated by the transpiration rate determined gravimetrically divided by the difference between soil and leaf water potential. Values are means ±SD of five replicates. Different letters indicate a significant difference (*P*<0.05).

Under control conditions, no change in K_plant_ was observed to occur due to the presence or absence of silicon treatment. Under osmotic stress, however, a sharp decrease in K_plant_ was seen in the absence of silicon treatment, but the K_plant_ was 45% higher in silicon-treated seedlings than in untreated seedlings (Fig. 6).

### Root hydraulic conductance and root surface area

**Fig. 7.**
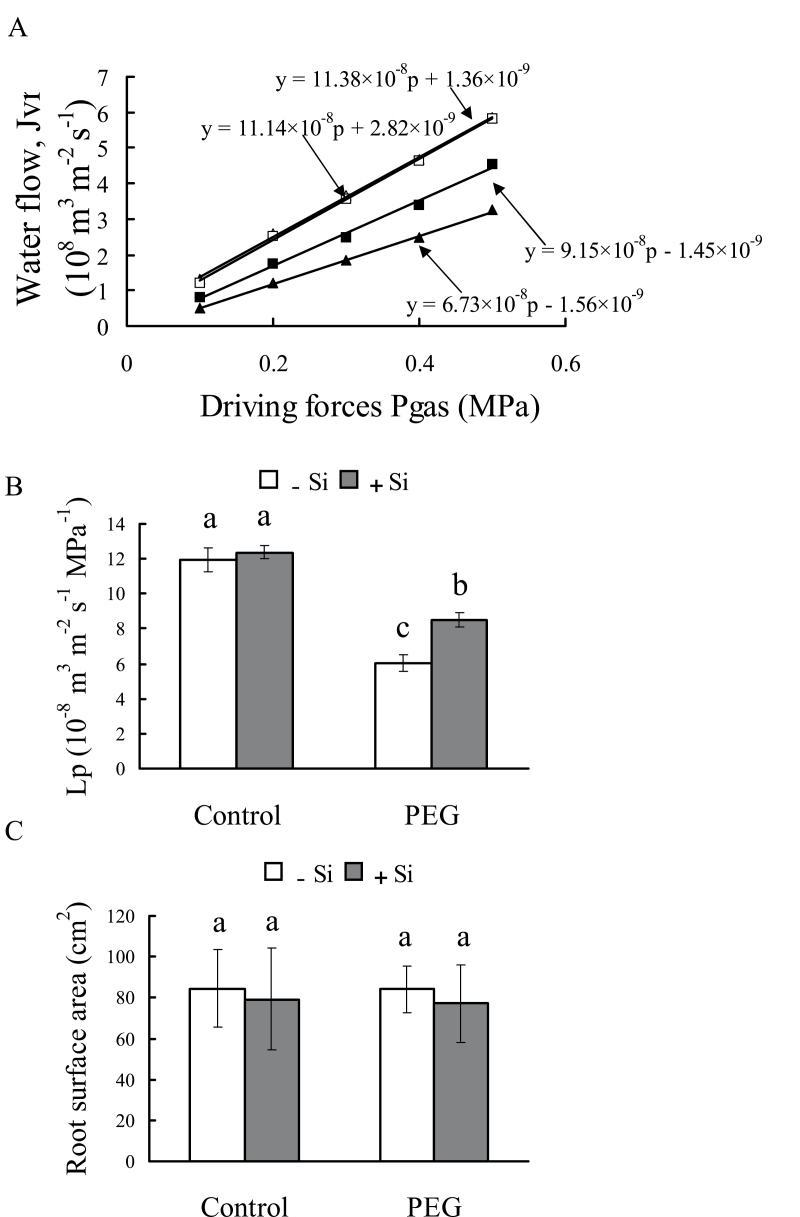
Effect of silicon application and osmotic stress on the root hydraulic conductance (Lp) in hydroponic culture. The whole-root system of seedlings under control (open symbols) or PEG treatment (filled symbols) with (squares) or without silicon (triangles) was cut off near the root base, leaving 4cm of mesocotyl, and inserted into the pressure chamber. For a given applied gas pressure, the volume exuded from the root system was plotted against time. (A) The slope of these relationships referred to unit root surface area was denoted as the Lp. (B) The means ±SD are shown. (C) The root surface used to calculate the Lp. Values are means ±SD of five replicates. Different letters indicate a significant difference (*P*<0.05).

The Lp was not affected by silicon application under control conditions (Fig. 7A, B), but it was affected under osmotic stress, where Lp decreased by only 31% in silicon-treated seedlings compared with 50% in untreated seedlings. The root surface area, vessel diameter, and number were not affected by silicon application under either control or osmotic stress conditions (Fig. 7C; Supplementary Fig. S3 at *JXB* online).

### Transpiration rate in response to HgCl_2_

**Fig. 8.**
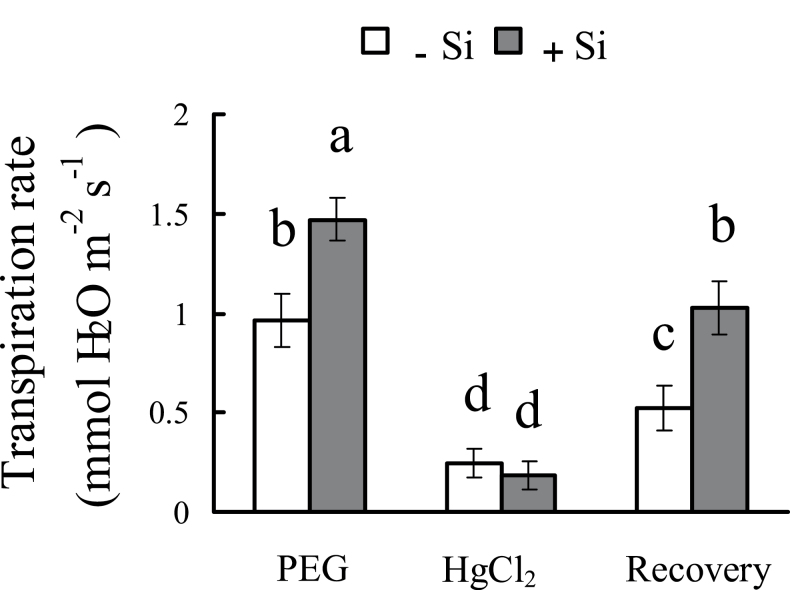
Effect of an aquaporin inhibitor (HgCl_2_) and an anti-inhibitor (dithiothreitol, DTT) on the transpiration rate with and without silicon application under osmotic stress. The transpiration rate was measured under osmotic stress before 50 μM HgCl_2_ (5min) was added to the culture solution, and the transpiration rate was measured again after HgCl_2_ was added. To investigate the recovery permitted by DTT, sorghum seedlings were exposed to, in quick succession, 50 μM HgCl_2_ (5min) and 5mM DTT (15min) before the transpiration rate was measured. Values are means ±SD of five replicates. Different letters indicate a significant difference (*P*<0.05).

In the presence of HgCl_2_, the transpiration rate decreased sharply, and the difference between osmotic-stressed plants with and without silicon application disappeared (Fig. 8). After a recovery induced by DTT, the transpiration was still higher in silicon-treated plants than in untreated plants. Similar results were found after treatment with NaN_3_: after NaN_3_ was added, the transpiration rates of plants with and without silicon treatment were not different (Supplementary Fig. S4 at *JXB* online).

### Expression of root aquaporin genes

**Fig. 9.**
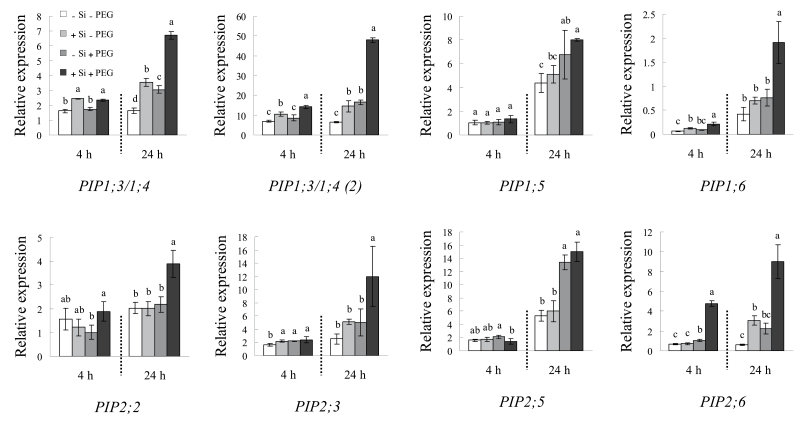
Effect of silicon application and osmotic stress on the expression levels of root *SbPIP* aquaporin genes. Root was sampled after 4h and 24h osmotic treatment both with and without silicon application. The relative expression was determined by qRT-PCR. Values are means ±SD of three replicates. Different letters indicate a significant difference (*P*<0.05).

As shown in Fig. 9, after 4h osmotic treatment, *SbPIP1;3/1;4*, *SbPIP1;3/1;4 (2)*, *SbPIP1;6*, *SbPIP2;2*, and *SbPIP2;6* were up-regulated by silicon application, especially *SbPIP1;3/1;4 (2)* and *SbPIP2;6*, whose expression levels were up-regulated by 1.64- and 4.55-fold, respectively. Only *SbPIP2;5* expression was decreased by silicon application under osmotic stress. After 24h PEG treatment, almost all of the *SbPIP* aquaporin genes were up-regulated, with the exception of *SbPIP1;5* and *SbPIP2;5*, whose expression was not affected by silicon.

### Leaf-specific conductivity of stem in sand culture

**Fig. 10.**
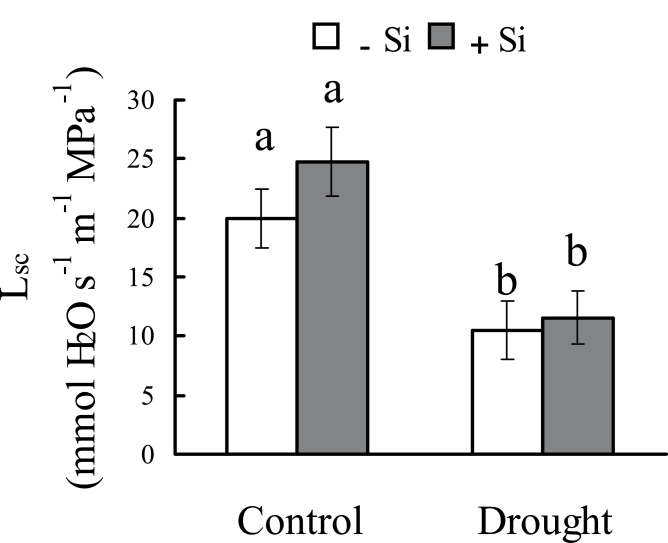
Effect of silicon application and water deficit on leaf-specific conductivity (L_sc_) of the stem in sand culture. After 3 d water deficit treatment, 40-day-old seedlings were used to measure the L_sc_ of the node. The second node from the top was used for L_sc_ measurement. Values are means ±SD of five replicates. Different letters indicate a significant difference (*P*<0.05).

The L_sc_ of the stem was remarkably decreased by water deficit stress, but it was not affected by silicon application under either control or water deficit conditions (Fig. 10). Furthermore, the vessel diameter and vessel number were also not affected by silicon in either control or water deficit conditions (Supplementary Fig. S5 at *JXB* online).

## Discussion

In general, plant biomass decreased significantly under osmotic stress; however, silicon application reduced the decrease in total dry weight of sorghum seedlings (Fig. 1), indicating that silicon is effective in improving sorghum resistance to osmotic stress. Hydraulic conductance regulation is fundamental to water regulation in plants; it can affect integrated responses such as stomatal movements or growth control under changing environmental conditions (Sutka *et al.*, 2011). Moreover, under drought stress, the ability to maintain a high photosynthetic rate is considered one of the most important drought resistance characteristics for plants (Cattivelli *et al.*, 2008). In this study, the photosynthetic rate was significantly decreased by water deficit stress (hydroponic and sand culture), but this decrease was greatly reduced by silicon (Fig. 3; Supplementary Fig. S2 at *JXB* online). The silicon concentration was found to be far higher in silicon-treated plants than in untreated plants (Fig. 2). These results clearly confirm that silicon application enhances sorghum resistance to osmotic stress. Previous studies have shown that changes in K_plant_ affect stomatal conductance and photosynthesis (Hubbard *et al.*, 2001). In this study, osmotic stress significantly decreases K_plant_, but silicon application reduced this decrease. Furthermore, the changes in photosynthetic rate and stomatal conductance that occurred in the presence of silicon were similar to the changes in K_plant_ that occurred under osmotic stress.

K_plant_ consists of leaf, stem, and root hydraulic conductance (Martre *et al.*, 2002). K_leaf_ tends to be very similar to leaf water potential (Brodribb *et al*., 2003). In the present study, the leaf water potential of sorghum with silicon was higher than that without silicon under osmotic stress. Therefore, it seems that silicon reduced the decrease in K_leaf_ under osmotic stress. Stem hydraulic conductance is represented by L_sc_ and controlled by the structure and size of vessels and also by the formation of embolisms (Lovisolo *et al.*, 1998). In this study, the stems of plants grown in hydroponic culture were not observed during the short growth period, but, in plants grown in sand culture, silicon application had no effect on vessel diameter, number of stems, or the L_sc_ of the stem (Supplementary Fig. S5 at *JXB* online). In addition, it is worth noting that L_sc_ was much higher than K_plant_, and that osmotic stress decreased K_plant_ greatly, but L_sc_ only slightly. This suggests that L_sc_ is not the limiting factor for water transport in sorghum seedlings under water deficit stress. The results support a previous study’s conclusion that the leaf and root contribute the major portions of whole-plant hydraulic resistance (Javot and Maurel, 2002).

Root hydraulic conductance (Lp) is usually the lowest within the liquid component of the soil–plant–air continuum (Vandeleur *et al.*, 2009). In the present study, the decrease in Lp was significantly reduced by silicon application under osmotic stress (Fig. 7A, B). Lp represents the root water uptake capacity and is determined by root surface, root anatomy, and root water permeability (Sutka *et al.*, 2011). In this study, no difference in root surface area or anatomic characteristics was found between seedlings with and without silicon application (Fig. 7C; Supplementary Fig. S3 at *JXB* online). This indicates that silicon did not affect water uptake by influencing root surface area or root anatomy. It has been suggested that water can move radially toward the xylem along three pathways: the apoplastic, symplastic, and transcellular pathways. The symplastic and transcellular pathways are collectively referred to as the ‘cell-to-cell’ pathway (Steudle, 2000a, *b*). Previous studies have suggested that, under drought stress, the cell-to-cell pathway plays an important role in water transport in the root and is driven by the osmotic gradient between the soil and root xylem sap (Javot and Maurel, 2002). In this study, the osmotic gradient was not affected by silicon application because the osmotic potential of root xylem sap was not changed by silicon under water deficit stress. In addition, it is worth noting that the osmotic potential of xylem sap (–0.16MPa) was higher than the osmotic potential of PEG solution (–0.20MPa). Thus, it is indicated that the osmotic driving force was not beneficial to water transport under osmotic stress in sorghum; a similar result has been found previously in maize (Hachez *et al.*, 2012). On the other hand, it has been reported that the ‘cell-to-cell’ pathway can be largely controlled by the activity of aquaporins, which respond relatively rapidly and reversibly, causing changes in Lp (Vandeleur *et al.*, 2009). In the present study, the decrease in Lp is significantly reduced by silicon application under short-term osmotic stress (Fig. 7A). These results suggest that aquaporins may be involved in the silicon-improved Lp under osmotic stress.

The participation of aquaporins was next tested by HgCl_2_ application. Under osmotic stress, most of the transpiration was repressed by HgCl_2_, and the difference between plants with and without silicon disappeared (Fig. 8). After HgCl_2_ treatment, the transpiration rate was inhibited by 88% in silicon-treated seedlings and by 73% in those which were not silicon treated. These results support the idea that the activity of aquaporins is involved in the silicon-improved Lp that occurs under osmotic stress, and that silicon application may promote the activity of aquaporins. These results also confirm that the cell-to-cell pathway plays a major role in overall water uptake under osmotic stress conditions in this study. Numerous studies have observed that plant up-regulation of aquaporin genes is beneficial to plant drought resistance (Lian *et al.*, 2004; Dugas *et al.*, 2011; Hachez *et al.*, 2012). In this study, the expression of several *SbPIP* genes under osmotic stress was increased 2- to 4-fold by silicon compared with untreated plants.

It is worth noting that in this study, the transpiration rate was 51% higher in silicon-treated plants than in those without silicon treatment under osmotic stress, and a similar tendency was also found in K_plant_ and Lp, which were 45% and 41% higher, respectively, in silicon-treated plants than in those without silicon treatment. The similar extent of the changes in those indexes that were induced by silicon under osmotic stress supported that silicon can regulate water uptake through improving the Lp, which was ascribed to up-regulation of aquaporins. The degree of change was slightly higher in transpiration rate than in Lp, suggesting that the high K_leaf_ in silicon-treated seedlings may also have a small influence on the transpiration rate.

Taking all the results together, this study suggests that silicon enhances sorghum resistance to water deficit osmotic stress through regulating Lp, which is decreased to a lesser extent by up-regulation of aquaporin gene expression under short-term osmotic stress. Based on these results, the mechanism by which silicon increases plant resistance to osmotic stress can be speculated. Under osmotic stress, high silicon concentrations in plants may trigger the up-regulation of aquaporin activity through affecting several stress signalling pathways. The up-regulation of aquaporin activity could lead to a reduction in the decrease in Lp and K_plant_, which was beneficial to water uptake and to keep the photosynthetic rate high, thus leading to enhancement of sorghum resistance to osmotic stress. In other species, such as rice, maize, and cucumber, silicon improves drought resistance through decreasing the transpiration rate, but whether aquaporins are involved in improved drought resistance in these species has not yet been resolved. To the authors’ knowledge, this is the first report to demonstrate that aquaporins are involved in silicon-induced plant resistance to osmotic stress. Furthermore, the results of this study also support the idea that silicon can act as a modulator that participates in the response to osmotic stress.

## Supplementary data

Supplementary data are available at *JXB* online.

Figure S1. Effects of silicon application and osmotic stress on transpiration rate in hydroponic culture.

Figure S2. Effects of silicon application and water deficit stress on photosynthetic rate (A), stomatal conductance (B), and transpiration rate (C) in sand culture.

Figure S3. Effects of silicon application and osmotic stress on vessel diameter (A) and number (B) in root.

Figure S4. Effects of an aquaporin inhibitor (NaN_3_) on transpiration rate with and without silicon application under osmotic stress.

Figure S5. Effects of silicon application and water deficit stress on vessel diameter (A) and number (B) in internodes in sand culture.

Supplementary Data

## Abbreviations:

DTT: dithiothreitol
K_plant_: whole-plant hydraulic conductance
Lp: root hydraulic conductance
L_sc_: leaf-specific conductivity
PIP: plasma membrane intrinsic protein
RWC: leaf relative water content.
