# Supplementary material for: Aquaporin-mediated increase in root hydraulic conductance is involved in silicon-induced improved root water uptake under osmotic stress in Sorghum bicolor L
Source: J Exp Bot. 2014 May 30;65(17):4747–56. doi: 10.1093/jxb/eru220 (PMC4144762; doi:10.1093/jxb/eru220)
Supplement: Supplementary Data [file supp_65_17_4747__index.html]

Aquaporin-mediated increase in root hydraulic conductance is involved in silicon-induced improved root water uptake under osmotic stress in Sorghum bicolor L — Aquaporin-mediated increase in root hydraulic conductance is involved in silicon-induced improved root water uptake under osmotic stress in Sorghum bicolor L. — Supplementary Data 

# Aquaporin-mediated increase in root hydraulic conductance is involved in silicon-induced improved root water uptake under osmotic stress in *Sorghum bicolor* L.

## Supplementary Data

Data files

**Files in this Data Supplement:**

- Supplementary Data - Supplementary Data
