# Supplementary material for: Aquaporin-mediated increase in root hydraulic conductance is involved in silicon-induced improved root water uptake under osmotic stress in Sorghum bicolor L
Source: J Exp Bot. 2014 May 30;65(17):4747–56. doi: 10.1093/jxb/eru220 (PMC4144762; doi:10.1093/jxb/eru220)
Supplement: Supplementary Data [file supp_eru220_jexbot106823_file001.pdf]

Title: Aquaporin-mediated increase in root hydraulic conductivity was involved with silicon-induced improved root water uptake under osmotic stress in *Sorghum bicolor* L.

Author: Peng Liu[1,*], Lina Yin[1, 2, 3,*], Xiping Deng[1, 2], Shiwen Wang[1, 2, †], Kiyoshi Tanaka[3], Suiqi Zhang[1, 2]

Supplementary figure S1

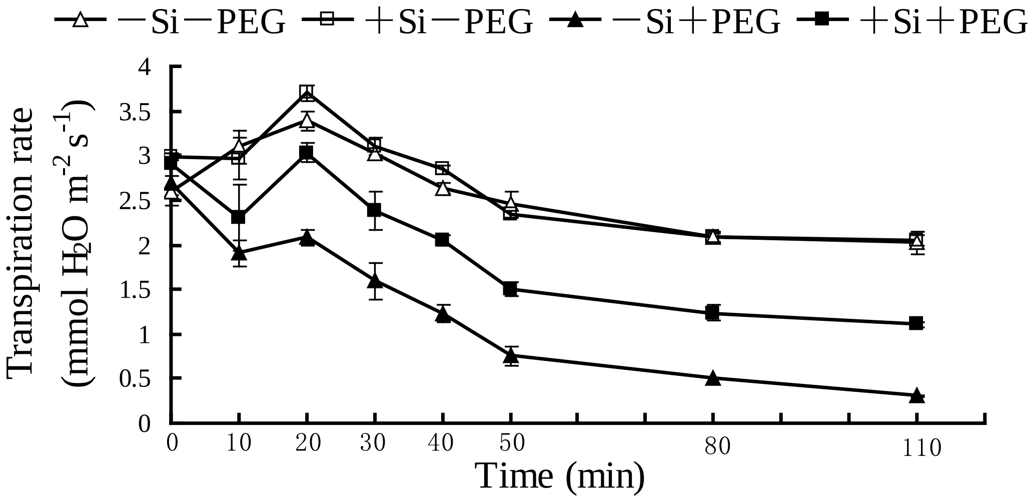

Fig.S1. Effects of silicon application and osmotic stress on transpiration rate in hydroponic culture. Transpiration rates of sorghum seedlings were determined gravimetrically from the initial of PEG treatment. There was no difference in transpiration rate between silicon application (square) and absent (triangle) under control condition (open symbols). The silicon reduce the decrease in transpiration rate was observed 20 min after the PEG treatment (closed symbols), and this phenomenon was lasted in the following 2 h. Values are means ± SD of five replicates.

Supplementary figure S2

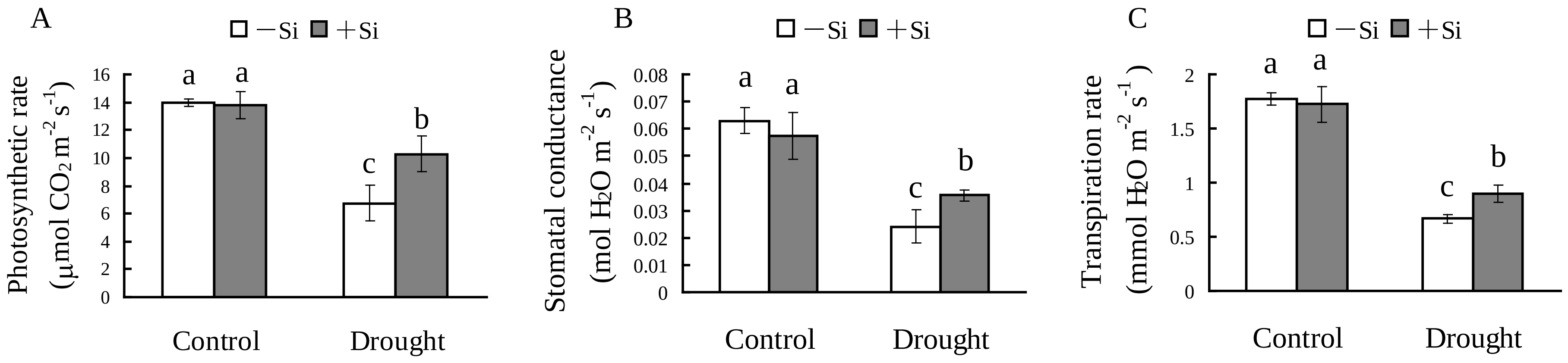

Fig.S2. Effects of silicon application and water deficit stress on photosynthetic rate (A), stomatal conductance (B) and transpiration rate (C) in sand culture. After 2 d of stopping watering, soil water content was down to 0.03 g g^{-1} and was kept for another day. Then new fully expanded leaf from the individual plant was used for measuring photosynthetic parameters by a portable photosynthesis system (Li-6400; LI-COR Inc., NE, USA) between 10:00 and 13:00. The changes of these parameters were similar with the results obtained under the hydroponic culture. Under control condition, these photosynthetic parameters were not affected by silicon. In contrast, under water deficit stress, silicon application significantly reduced the water deficit-induced decrease of these parameters. Values are means ± SD of five replicates. Different letters indicate significant difference ($P < 0.05$).

Supplementary figure S3

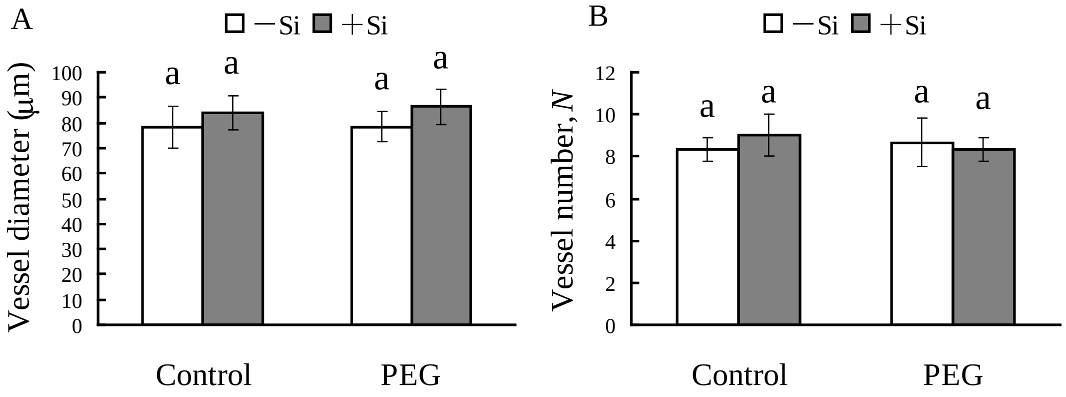

Fig.S3. Effects of silicon application and osmotic stress on vessel diameter (A) and vessel number (B) in root. The first nodal root was excised to investigate the root anatomic characteristics. Free-hand cross-sections of nodal root were pictured by optical microscope (BX51, Olympus) and analyzed with Image-Pro-Express Chinese 6.0. The vessel diameter and number were also not affected by silicon under both control and osmotic stress. Values are means ± SD of five replicates. Different letters indicate significant difference ($P < 0.05$).

Supplementary figure S4

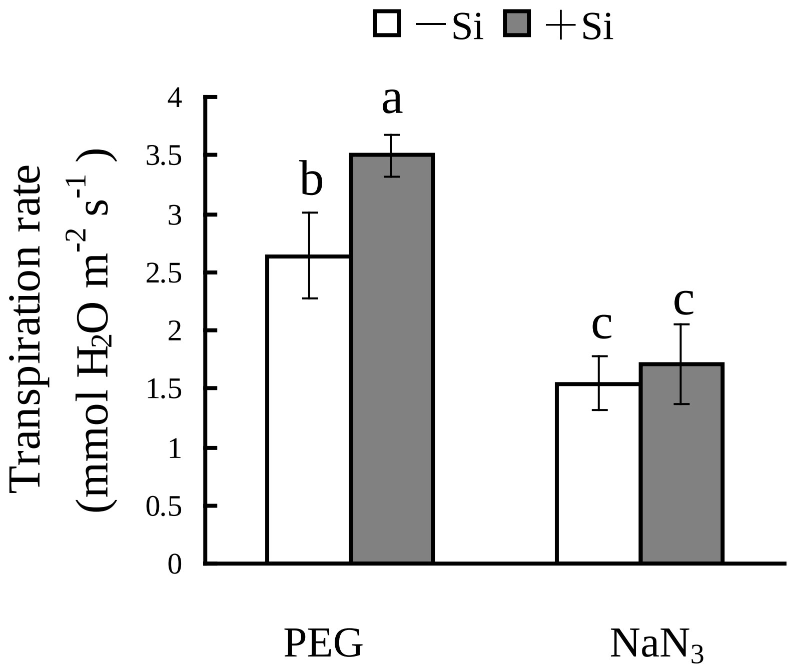

Fig.S4. Effect of aquaporin inhibitor (NaN_3) on transpiration rate with and without silicon application under osmotic stress. The 12 d seedlings were used for measurement. The transpiration rate was measured after 2 h osmotic stress before NaN_3 was added, and the transpiration rate was measured again after treated by 1 mM NaN_3 for 20 min (Sutka et al., 2011). Under osmotic stress, the transpiration rates of seedlings with silicon application were significantly higher than that without silicon application. However after NaN_3 treatment, the transpiration rates were decreased to the same level. Values are means ± SD of five replicates. Different letters indicated significant difference ($P < 0.05$).

Supplementary figure S5

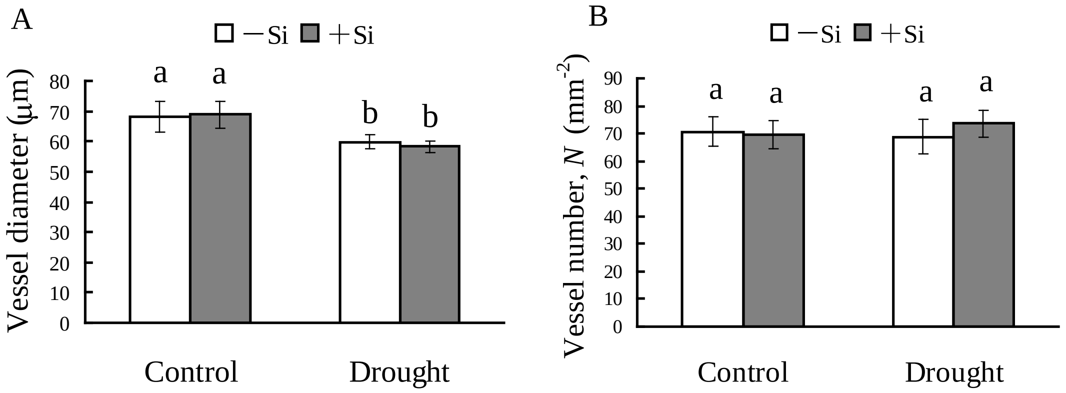

Fig.S5. Effects of silicon application and water deficit stress on vessel diameter (A) and number (B) in internode in sand culture. The internode between the second and third node was used for investigating the vessel diameter and number. Free-hand cross-sections of internode were pictured by optical microscope (BX51, Olympus) and then the pictures were analyzed with Image-Pro-Express Chinese 6.0. Although the diameter was decreased under water deficit stress, it was not affected by silicon under both control and water deficit conditions. Vessel number was also not affected by silicon both under control and water deficit conditions. Values are means ± SD of seven replicates. Different letters indicate significant difference ($P < 0.05$).
